# Novel methods for quantifying individual host response to infectious pathogens for genetic analyses

**DOI:** 10.3389/fgene.2012.00266

**Published:** 2012-12-14

**Authors:** Andrea B. Doeschl-Wilson, Steve C. Bishop, Ilias Kyriazakis, Beatriz Villanueva

**Affiliations:** ^1^The Roslin Institute and Royal (Dick) School of Veterinary Studies, University of EdinburghEdinburgh, UK; ^2^School of Agriculture, Food and Rural Development, Newcastle UniversityNewcastle upon Tyne, UK; ^3^Departamento de Mejora Genética Animal, INIAMadrid, Spain

**Keywords:** infectious disease, host–pathogen interaction, tolerance, resistance, random regression, dynamical system, infection dynamics, breeding for disease resistance

## Abstract

We propose two novel approaches for describing and quantifying the response of individual hosts to pathogen challenge in terms of infection severity and impact on host performance. The first approach is a direct extension of the methodology for estimating group tolerance (the change in performance with respect to changes in pathogen burden in a host population) to the level of individuals. The second approach aims to capture the dynamic aspects of individual resistance and tolerance over the entire time course of infections. In contrast to the first approach, which provides a means to disentangle host resistance from tolerance, the second approach focuses on the combined effects of both characteristics. Both approaches provide new individual phenotypes for subsequent genetic analyses and come with specific data requirements. In particular, both approaches rely on the availability of repeated performance and pathogen burden measurements of individuals over the time course of one or several episodes of infection. Consideration of individual tolerance also highlights some of the assumptions hidden within the concept of group tolerance, indicating where care needs to be taken in trait definition and measurement.

## Introduction

Resistance and tolerance to infectious pathogens are important characteristics of livestock to counteract the potential detrimental impact of pathogens on animal health and production. Host resistance refers to the ability to reduce pathogen replication, in the broadest sense, whereas tolerance refers to the ability to reduce the impact of pathogens on host performance without necessarily affecting pathogen burden. In order to target these characteristics for genetic improvement, they need to be quantifiable. Host resistance is generally quantified by a measure of infection severity such as within-host pathogen burden (e.g., viral or bacterial counts or parasite density). Tolerance may be quantitatively defined as the change in host performance with respect to change in pathogen burden (e.g., Simms, 2000), where performance may refer to any trait relevant for production or reproduction (e.g., growth rate, feed intake, or litter size).

As outlined in our companion paper (Doeschl-Wilson et al., 2012), although conceptually tolerance is defined at the individual level (Simms, 2000; Schneider and Ayres, 2008), empirical tolerance estimates have only been obtained at the level of groups of (related) individuals. In particular, group tolerance estimates are usually derived using analysis of covariance (ANCOVA) or random regression approaches, where performance measures of infected group members are regressed against their respective pathogen burden at a given time post infection (e.g., Simms and Triplett, 1994; Mauricio et al., 1997; Råberg et al., 2007; Kause, 2011). Whilst these approaches may provide useful evidence on whether genetic variation in tolerance exists, the resulting group estimates have several disadvantages that render them not ideal for genetic studies, for the three reasons outlined below.

The first issue is that these estimates rely on the underlying assumption that all animals have been exposed to, or infected with, the same dose/type of pathogens at the same time. This assumption is unlikely to hold in field conditions and it raises the question of whether reliable group tolerance estimates can be obtained from field data (Doeschl-Wilson et al., 2012), which are the primary data source for quantitative genetic analyses of disease traits (Bishop and Woolliams, 2010).

Secondly, group tolerance estimates, which are usually obtained from cross-sectional measures (i.e., measures taken at one time point during the infection) (Simms and Triplett, 1994; Mauricio et al., 1997; Råberg et al., 2007; Ayres and Schneider, 2012), may poorly represent the overall impact of infection on host performance over the entire time course of infection. Consider for example two families with equal average resistance, i.e., the same pathogen burden profiles. Assume however that members of family A have a significantly greater ability to prevent tissue damage inflicted by pathogens than members of family B but members of family B have developed more efficient recovery mechanisms (e.g., tissue repair mechanisms) than those belonging to family A. Thus, ANCOVA or random regression may indicate significant family differences in tolerance depending on when during the infection process the performance and pathogen burden records were taken. Based on early measurements family A would be selected as more tolerant, whereas based on late measurements members of family B may have emerged as favorable selection candidates. The example illustrates that the effects of host tolerance may vary throughout the infection as a result of different mechanisms acting at different stages. Measurements taken at different stages of the infection may therefore give rise to different tolerance estimates and hence also to different estimates of genetic variance in tolerance. Biased estimates of genetic parameters may therefore arise if these dynamic changes are neglected. In principle, group tolerance estimates that describe the impact of infection on performance over the entire time course of one or several episodes of infection could be obtained by using cumulative measures of performance and pathogen burden over time rather than cross-sectional measures, as suggested by Ayres and Schneider (2012). However, cumulative measures would require repeated measurements of both host performance and pathogen burden on infected individuals, in addition to measurements of host performance in the absence of pathogen challenge (or in less pathogenic environments), as established in our companion paper (Doeschl-Wilson et al., 2012). In summary, in order to obtain a reliable estimate of tolerance at the *group* level a multitude of measurements at the *individual* level would be needed. This seems a disproportionately large effort for the limited genetic gain that can be achieved with group selection.

Finally, from an animal breeding perspective, another major drawback of group rather than individual tolerance is that selection accuracy and therefore response to selection is limited if phenotypes are specified at the group level (Falconer and Mackay, 1996). Furthermore, group phenotypes do not take advantage of the benefits of recent advances in high throughput genomics for dissecting host responses to infectious pathogens and increasing the accuracy of selection. For instance, increased availability of dense Single Nucleotide Polymorphism (SNP) chips in most livestock species has facilitated genome-wide prediction for obtaining accurate breeding values. For example, SNP markers whose effects have been calibrated in individuals that are both genotyped and measured for the trait of interest may then be used to obtain estimated breeding values for animals that are genotyped but not phenotyped. Thus, with genome-wide evaluations, obtaining phenotypes for disease traits (a difficult task in most cases) can be avoided for at least some generations before marker effects need to be re-estimated. Having only group tolerance phenotypes available implies that information arising from within-group variation cannot be exploited in the first step, thus sacrificing potential accuracy of selection.

In summary, the availability of individual phenotypes of both host resistance and tolerance would be highly desirable for quantifying infection severity and impact on production over the time course of one or several episodes of infection. Both resistance and tolerance are defined and expressed at the level of individuals. It is the limitation of current statistical approaches that restrict the estimation of host tolerance to the level of groups. In this article two novel approaches for estimating individual tolerance are proposed. Both approaches rely on the availability of repeated measurements of host performance and pathogen burden. The first approach is a direct extension of the methodology for estimating group tolerance to the level of individuals, and provides a means to disentangle host resistance from tolerance. The second approach aims to capture the combined dynamic effects of individual resistance and tolerance over the entire time course of infections in terms of infection severity and impact on performance.

## Extending the statistical framework from group tolerance to individual tolerance

The well-established definition of tolerance as the change in performance with respect to changes in pathogen burden (Simms, 2000) implies that to quantify tolerance at the level of individuals, repeated measurements of host performance and pathogen burden from individual animals would be needed. In other words, individual tolerance can only be quantified if within host pathogen burden changes over time, and both performance and pathogen burden can be measured repeatedly on each individual. Thus, traits that can only be measured once (e.g., carcass and survival traits) are not suitable for estimating individual tolerance. For repeatedly measurable performance traits (e.g., growth rate, milk yield, litter size), individual tolerance may describe how an individual's performance is affected over the time course of one infection, or over several episodes of infections, depending on when measurements are taken.

### Mathematical framework for individual tolerance

Group tolerance is typically inferred by regressing measurements of host performance (usually collected at a specific time point post infection) against corresponding measurements of pathogen burden for individual group members (e.g., Simms, 2000; Råberg et al., 2007; Kause, 2011). The framework can be extended to estimate tolerance of individuals by replacing cross-sectional measurements of multiple individuals by multiple repeated measurements per individual. However, as a consequence of using repeated measurements taken at different time points, time would need to be included explicitly when describing the relationship between performance and pathogen burden. In other words, instead of fitting the linear model *y* = *y*0 + *bPB* currently used to estimate group tolerance, which corresponds to a snapshot in time, a time-dependent model would need to be used:

(1) y(t) = y0(t)−b(t)PB(t)

where *y*0(*t*) denotes host performance at time *t* in a pathogen-free environment and *b*(*t*) describes the effect of pathogen burden on host performance at time *t*. Several implications of using the time-dependent model (1) for estimating individual tolerance should be pointed out. Firstly, taking the first order derivative of *y*(*t*) with respect to time, i.e.,

(2) $$\frac{dy}{dt}=\frac{dy0}{dt}-\frac{db}{dt}PB-b\frac{dPB}{dt}$$

illustrates that a change in performance of an infected individual can be the result of three different causes:

1. A change in host performance over time not related to pathogen challenge ($\frac{dy0}{dt}$), i.e., a change that would also occur if the individual was not infected.
2. A change in the impact of a unit pathogen dose on host performance ($\frac{db}{dt}PB$), i.e., host-induced change in pathogen virulence over the time course of infection.
3. A change in host performance associated with changes in within-host pathogen burden ($b\frac{dPB}{dt}$).

Only the last two components, i.e., those including expressions of *b*(*t*), are associated with tolerance mechanisms.

Secondly, Equation (2) reveals that in order to obtain accurate estimates of tolerance effects, natural temporal variations in host performance would need to be accounted for. It also reveals that tolerance, which is mathematically defined as $\frac{dy}{dPB}$ may change over time. This becomes evident from Equation (2) after expressing $\frac{dy}{dPB}$ as

(3) $$\frac{dy}{dPB}=\frac{\frac{dy}{dt}}{\frac{dPB}{dt}}$$

Substituting Equation (2) into (3) shows that the mathematical definition of tolerance as the incremental change in performance with respect to pathogen burden, i.e., $\frac{dy}{dPB}$ is generally not the same as *b*(*t*). Indeed, $\frac{dy}{dPB}$ is only equal to *b*(*t*) if both *y*0(*t*) (performance in the absence of pathogen challenge) and *b*(*t*) (impact of pathogen burden on host performance) are constant over time (so that their derivatives with respect to time are 0). However, since $\frac{dy}{dPB}$ encompasses also changes in host performance not related to pathogen burden, tolerance may be more appropriately represented by the term *b*(*t*). This is also consistent with the classical definition of tolerance as the slope *b* in Equation (1).

### Statistical framework for estimating individual tolerance

Random regression models have proved to be a useful framework for estimating tolerance of families of individuals (Kause, 2011; Kause and Ødegård, 2012). In particular, Kause (2011) demonstrated that the tolerance *bj* of a group *j* can be estimated as the regression slope of the linear model

(4) $$y_{ij}=y0_{ij}-b_{j}PB_{ij}+e_{ij}$$

where *y*_*ij*_ and *PB*_*ij*_ refer to host performance and pathogen burden of individual *i* in group *j* obtained at a fixed time post infection, respectively. It has been also established that in order to obtain unbiased group tolerance estimates *bj*, estimates of host performance *y*0_*ij*_ in a pathogen-free environment would be required (Kause, 2011; Doeschl-Wilson et al., 2012). Such estimates may be obtained simply from having group members in environments differing in pathogen challenge.

The statistical random regression framework can be extended to estimate tolerance of individuals by replacing the cross-sectional measurements *y*_*ij*_ and *PB*_*ij*_ of multiple individuals in Equation (4) by multiple repeated measurements per individual. Thus, the time-dependent mathematical model (1) relating individual host performance at time *t* to the corresponding pathogen burden can be represented as a statistical repeated measurement model as follows:

(5) $$y_{ik}=y0_{ik}-b_{ik}PB_{ik}+e_{ik}$$

where *y*_*ik*_ and *PB*_*ik*_ are the performance and pathogen burden of individual *i* measured at discrete time *t*_*k*_, respectively, *y*0_*ik*_ is the performance of individual *i* at *t*_*k*_ in a pathogen-free environment (assumed to be known), *b*_*ik*_ represents the impact of pathogen burden at time *t*_*k*_ on host performance (to be estimated), and *e*_*ik*_ is the individual error at *t*_*k*_ (to be estimated).

In order to obtain estimates for the tolerance parameter *b*_*ik*_ further information or assumptions are required. Firstly, *y*0_*ik*_ is assumed to be known. However, as a host cannot be simultaneously infected and non-infected, measures of *y*0 throughout the time period of infection do not exist and would thus need to be inferred from available measurements taken at different times. Thus, accurate tolerance estimates at the level of individuals can only be obtained for performance traits and time periods over which host performance in the absence of pathogens is known [e.g., weight loss in mature animals as used by Råberg et al. (2007)] or can be inferred based on measurements prior to infections (e.g., projected growth trajectory or milk yield).

Secondly, *b*_*ik*_ needs to be either assumed as constant over time (i.e., *b*_*ik*_ = *b*_*i*_ for all *t*_*k*_) or expressed in a form that can be included in the above model. Note that temporal variation in *b* corresponds to temporal variation in the impact that a unit dose of pathogens inflicts on host performance, i.e., temporal variation in host-induced pathogen virulence. Thus, a constant value *b*_*i*_ is justified in cases where the impact of a given pathogen burden does not change over time. This may be the case for example if the time interval over which tolerance is to be estimated is relatively short or if tolerance estimates refer to similar time points post infection during successive episodes of infection for individuals with no lasting immunity. The latter may apply to nematode infections in peri-parturient ewes or to mastitis in lactating cows. However, the assumption is less likely to hold if tolerance is to be estimated over the entire time course of one infection, as changes in the host immune response over time (e.g., mechanisms related to damage prevention or repair) would generally correspond to host-induced changes in pathogen virulence. However, in this case, i.e., when changes in pathogen virulence cannot be ignored, a simple expression for *b*_*ik*_ may be used. For example, adopting the principle of Occam's razor, *b*_*ik*_ in Equation (3) may be represented by

(6) $$b_{ik}=b_{i}(t_{k})=b_{0i}-v_{i}t_{k}$$

where *b*_0*i*_ describes the change in performance of individual *i* due to change in pathogen burden and *v*_*i*_*t*_*k*_ with constant rate parameter *v*_*i*_ describes the change in the impact of a unit dose of pathogens on performance. For parameter estimation, it would be more convenient if data were obtained from challenge experiments, where *t*_*k*_ refers to time post infection. Substitution of Equation (6) into (5), and appropriate specifications of variance and covariance structures for the individual parameters, subsequent measurements, and residuals, in principle yields individual estimates for the tolerance parameters *b*_0*i*_ and *v*_*i*_, which refer to different tolerance components. These estimates would constitute the phenotypes for subsequent genetic analyses. Exact data requirements for such an approach and properties of the obtained estimates have yet to be explored.

## A new framework for quantifying individual resistance and tolerance using dynamical systems theory

The statistical framework described above for quantifying individual (and group) tolerance relies on a number of stringent assumptions and measurement requirements. In particular, regression models assume a specific (direct) relationship between host performance and pathogen burden that can be formulated by a simple (often linear) mathematical function. This function may be a poor representation of the underlying biological processes affecting infection severity and impact on performance. For example, reduction in host performance may be caused by immune processes (Coop and Kyriazakis, 1999; Ayres and Schneider, 2012; Glass, 2012) rather than directly by pathogens. Although this was captured to some extent by Equation (6), one may question whether a statistical model that assumes a direct (and often linear) relationship between pathogen burden and performance can yield reliable tolerance estimates.

Furthermore, both resistance and tolerance usually relate to immunological processes (e.g., blocking pathogen entry in host target cell or reducing pathogen reproduction rate and preventing or repairing tissue damage) which are highly dynamic and often temporary. A conventional statistical framework may not be the most appropriate means of capturing these dynamic aspects. Instead, an alternative mathematical approach tailored toward dynamic processes may be better suited to capture the information revealed by repeated measurements of host pathogen burden and performance over time. This is outlined below.

### Performance vs. pathogen burden trajectories

**Figure 1:**
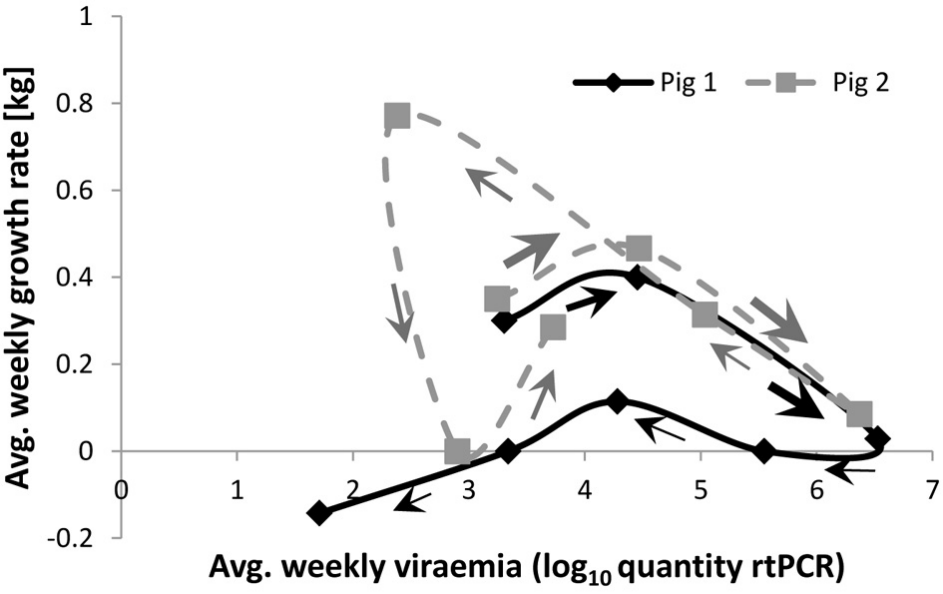
**Performance (average weekly growth rate) *vs.* pathogen burden (average weekly viraemia) trajectories for two individual pigs infected with the same challenge dose of a virulent PRRS virus strain.** Arrows indicate the direction of growth rate-viraemia plots over time. The size of the arrows crudely reflect the speed at which the trajectory progresses. Data are courtesy of the PRRS Host Genetics Consortium—for more information about data acquisition and experimental design see Rowland et al. (2012).

**Figure 2:**
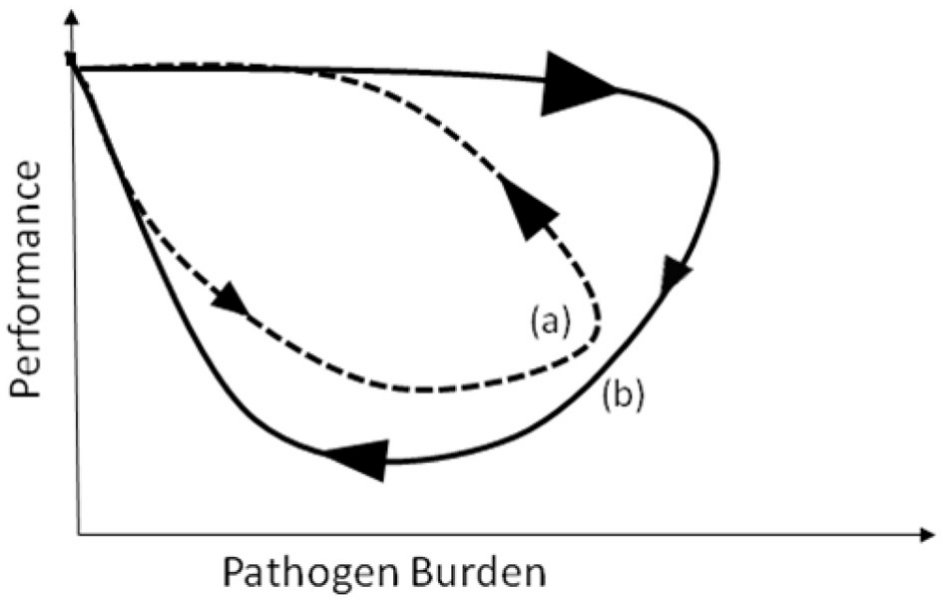
**Schematic figure representing pathogen burden–performance trajectories for two individuals (a) and (b) with different resistance and tolerance mechanisms.** The direction and size of the arrows indicate the direction and velocity at which trajectories evolve over time.

Scatter plots of individual performance *vs.* pathogen burden are fundamental for quantifying group tolerance (e.g., Simms, 2000; Råberg et al., 2007, 2009; Doeschl-Wilson et al., 2012). Adapting these plots to the level of an individual, i.e., by plotting the individual's performance *vs*. pathogen burden measurements collected at different time points, generates a trajectory in the pathogen burden–performance space (Figures 1, 2). This trajectory can reveal important information on how the individual's resistance and tolerance mechanisms interact over time. Consider for example the growth rate–pathogen burden trajectories of the two pigs depicted in Figure 1. These trajectories were generated based on repeated measurements of body weight and virus load collected over a period of 42 days after challenging the pigs with a given dose of the Porcine Reproductive and Respiratory Syndrome virus [experiment described by Rowland et al. (2012)]. The trajectory of pig 1 shows that the pig apparently manages to clear the virus within the observation period of 42 days, but suffers a long-term reduction in growth (growth rate at the end of infection is negative). In contrast, the trajectory of pig 2 indicates that the pig did not manage to clear the virus within the 42 day observation period—in fact it seemingly experienced viral re-activation. Nevertheless its growth rate at the end of the observation period was similar to that at the beginning and remained positive throughout the entire observation period. Furthermore, closer inspection of the trajectory corresponding to pig 1 reveals that reduction in growth was primarily associated with two distinct phases of the infection, i.e., the phase when viraemia levels rapidly increase toward peak levels and the recovery phase when viraemia has almost been cleared. The trajectory thus suggests that the overall impact of infection on performance may be partly associated with pathogen replication at the early stage and may be partly due to immune response mechanisms associated with the recovery process. Pig 2 has initially a similar trajectory to that of pig 1. However, its growth rate increased immediately once viraemia levels started to fall post peak viraemia and declined again after the re-activation of virus replication. Thus, for pig 2 changes in performance directly mimicked changes in pathogen burden.

As indicated by the arrows in Figure 1, trajectories are not only characterized by their shapes but also by the direction and speed at which pathogen burden—performance measures progress over time. These can reveal information about how host resistance and tolerance mechanisms interact during the time course of infection. To illustrate this more clearly, consider the schematic trajectories depicted in Figure 2. Both are closed loops indicating full recovery of the host in terms of pathogen elimination and restoration of host performance. Nevertheless, the reverse direction and different sizes of the arrows indicate substantial differences between the hosts in their response to pathogen challenge. For host (a) pathogen burden increases initially slowly (indicated by a small arrow) causing a gradual decrease in performance. This could be considered as moderate resistance and low tolerance at the early stage of the infection. Once pathogen burden peaks, host immune mechanisms counteract pathogen replication and damage leading to fast recovery (indicated by a large arrow). Thus, for host (a) changes in performance are a direct consequence of changes in pathogen burden. In contrast, host (b) experiences initially a rapid increase in pathogen burden (indicated by a large arrow), but its performance is not affected at all during this initial phase of the infection (i.e., low resistance but high tolerance at the early stage of infection). Performance, however, declines after pathogen levels start to decrease, indicating that the immune response rather than the actual pathogens are leading to the reduction in performance. Recovery is initially slow, but accelerates at the later stage of infection when performance levels also recover (i.e., high resistance and high tolerance at the late stage of infection). This situation could arise is different sets of immune mechanisms dominate at different stages of infection, comparing the two hosts.

### A new classification of hosts according to their trajectories

Trajectories may be considered to illustrate the dynamic interactions of resistance and tolerance mechanisms over time. Thus, a host may be characterized by its trajectory rather than by its resistance and tolerance, which are assumed static. Consequently, instead of aiming to improve host resistance or tolerance, one may aim to achieve an optimal trajectory in the pathogen burden–performance space. This would correspond to breeding for a combined optimal balance of tolerance and resistance mechanisms. But how could this be achieved in practice?

**Figure 3:**
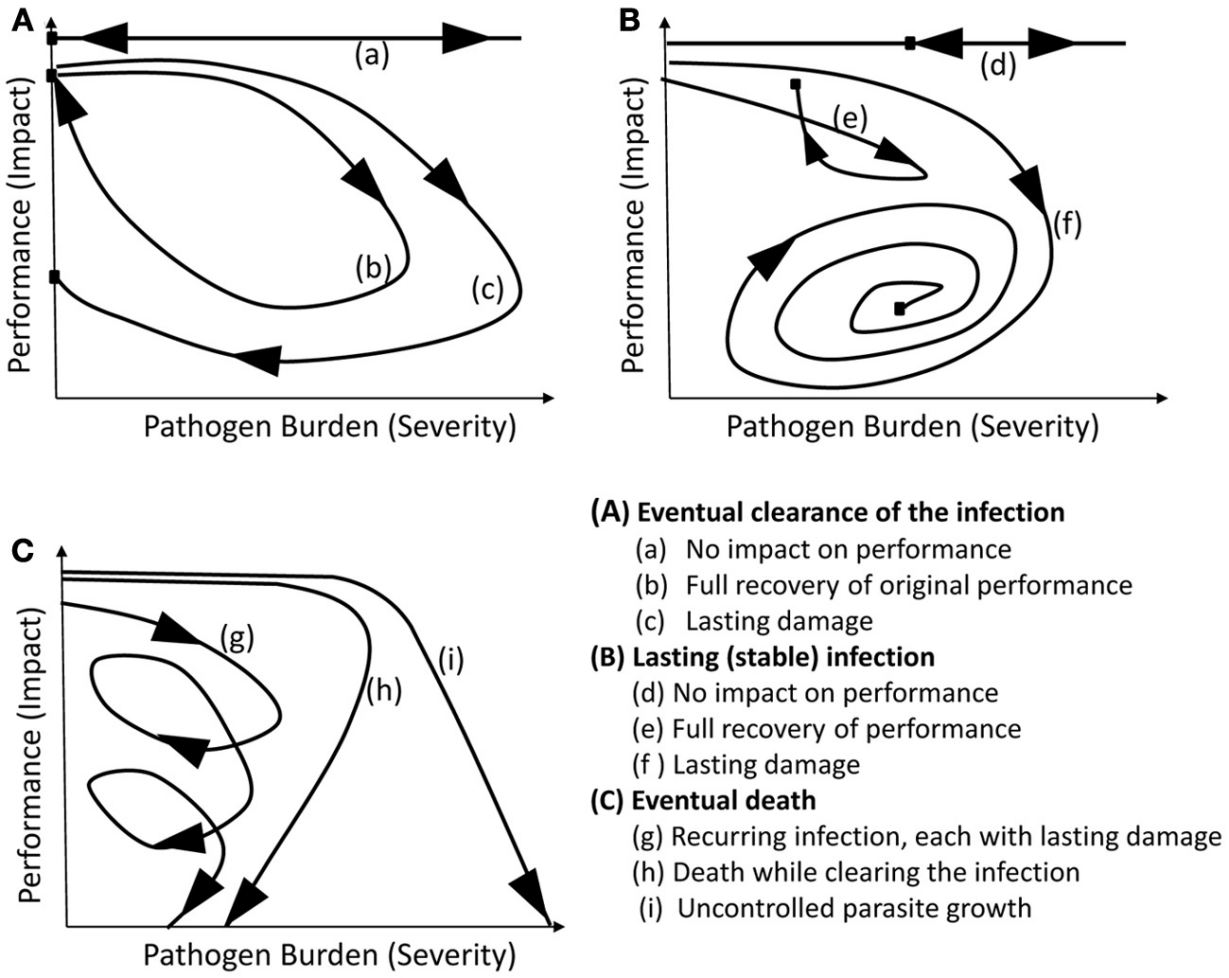
**Schematic figure of the nine trajectory archetypes.** The three graphs broadly correspond to different resistance categories, while the three individual curves in each graph broadly correspond to different tolerance categories. For further explanation see text.

Schneider (2011), after investigating the shapes of “personalized health curves” (equivalent to the pathogen burden–performance trajectories described here) corresponding to various well-studied infections in humans, concluded that most pathogenic and mutualistic host–pathogen interactions can be represented by one of only a few existing archetypical curves. Adapted to the resistance-tolerance context for the majority of livestock diseases, nine major classes of trajectory categories emerge as follows: individual host trajectories may first be classified according to the outcome in terms of infection severity, distinguishing broadly between eventual clearance of pathogens, long-term persistence, and death (illustrated by graphs A, B, C, respectively in Figure 3). Within each of these three categories, one may then further classify trajectories according to the long-term impact of infection on host performance. Thus, for infections leading to eventual clearance or long-term persistence, one may distinguish between hosts that experience little or no impact on performance, and those that suffer a temporary or persistent reduction in performance, respectively (illustrated by the different trajectories in Figures 3A,B). Similarly, if the final outcome is death, one may categorize trajectories according to whether death was caused either through cumulative damage caused by recurrent episodes of disease outbreaks, each leaving long-term damage, or while clearing the pathogens, or as a direct consequence of uncontrolled pathogen replication. All host–pathogen type interactions will fall within one of these nine categories although the actual shapes of individual trajectories within each category may differ considerably from each other and from those shown in Figure 3. Furthermore, depending on the type of pathogens and the host population, only a subset of these nine categories may be realized in practice. In any case, this framework implies that the individual scatter plots produced by data that are inherently noisy and incomplete could be characterized as one of nine types. As a first step in disease control one may thus aim to produce the best feasible trajectory type or look for genotypes associated with specific trajectory types.

### How to summarize the trajectories by quantitative measures for genetic analyses?

Having established the potential for pathogen burden–performance trajectories to describe impacts of infection on hosts, we are confronted with a number of further questions. Firstly, how does one generate complete individual trajectories with relatively limited measurements over time? In reality, measurements may only exist for some time points during infection, generating, at best, a fragment of a trajectory. For example, for the pigs in Figure 1, growth rates prior to infection were not recorded, making it difficult to infer whether infection led to long-lasting damage in growth. Secondly, is it possible to generate a complete a trajectory based on only few measurements over a restricted time period during an infection? Finally, and most importantly, in cases where all host responses appear to fall within the same trajectory type, how can one quantify individual trajectories and summarize the information into phenotypes for subsequent genetic analyses? The answers to these questions can be found by applying mathematical dynamical systems theory, as outlined below.

### Dynamical systems theory

In mathematical terms, the individual performance *vs*. pathogen burden trajectories may be referred to as trajectories in the *pathogen burden–performance phase space*. Phase space plots, in which individual trajectories are not only characterized by the shape of the curve, but also by the direction and velocity at which these curves evolve over time (indicated by the arrows in Figures 1–3), are commonly used to visualize and analyze the global behavior of dynamical systems (Katok and Hasselblatt, 1995). Indeed, these trajectories elicit system properties that might not otherwise be obvious, such as at what stage the infection is most damaging to the host and whether, when and to what extent recovery occurs.

**Figure 4:**
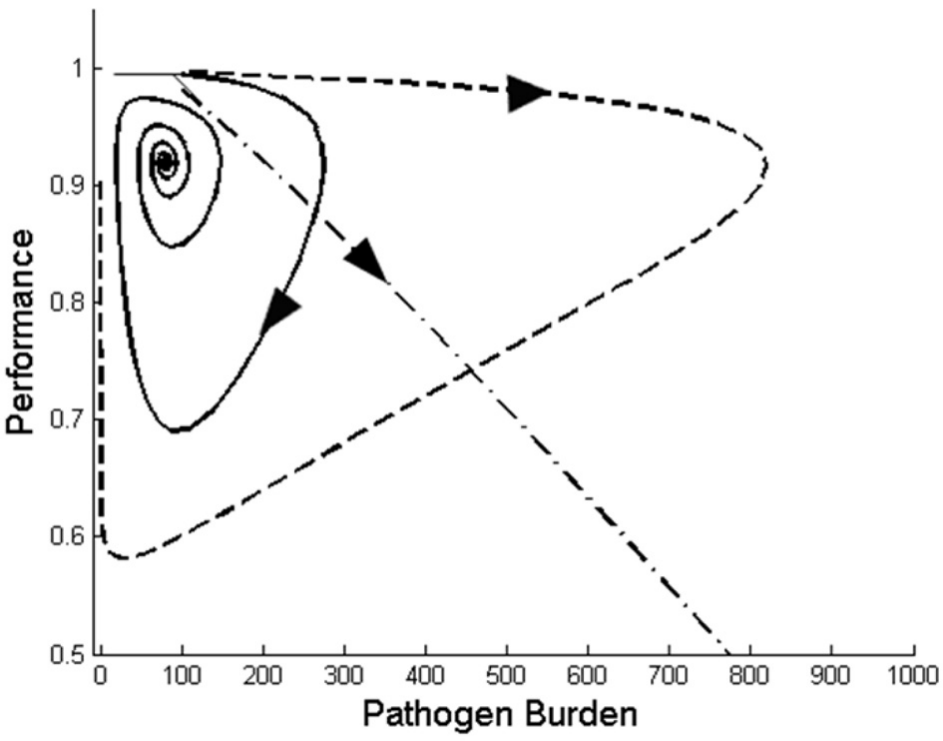
**Three different types of performance *vs*. pathogen burden profiles generated by the mathematical dynamical system (7) for different values of the model parameters.** The size of the arrows in this graph does not reflect the actual velocity.

In order to specify the individual trajectories over the entire time course of infection, and to reduce or remove noise from the data, it is advantageous to use mathematical models fitted to the data rather than the actual data for subsequent analyses. Mathematicians usually describe dynamical system by a set of differential equations. When adopting the dynamical systems theory to the context of host resistance and tolerance, it is therefore necessary to formulate mathematical expressions that describe the change in within-host change in pathogen burden and performance over time based on existing biological knowledge and available data. Thus, step 1 of this approach consists of plotting the (noisy, fragmented) performance *vs*. pathogen burden curves for the individuals in question, and step 2 consists of formulating an appropriate mathematical model that reproduces the essential features of these trajectories. For example, if the data suggest that changes of host performance are partly caused by the pathogen and partly by the immune response, one may start with a three-dimensional system of differential equations that describes changes in host pathogen burden (P) immune response (I) and performance (Y) over time:

(7) $$\begin{array}{l} \frac{dP}{dt}=\mu P-\kappa PI\\ \frac{dI}{dt}=\lambda \text{+}\rho I\frac{P}{P+\phi }-\delta I\\ \frac{dY}{dt}=-\beta \frac{dP}{dt}-\alpha \frac{dI}{dt} \end{array}$$

where μ denotes the replication rate of the pathogen within the host, κ is the rate at which the host immune eliminates the pathogen, λ and δ refer to the replacement and death rate of immune cells, respectively, ρ is the maximum per capita replication rate of the immune response, ϕ represents the parasite density at which the rate of growth of immunity is half maximal, and β and α describe the reduction in performance with respect to changes in pathogen burden and immune response, respectively. System (7) is an extension of the model described by Doeschl-Wilson et al. (2009). Although apparently simple, the model can generate a variety of trajectory types for different combination of model parameters (Figure 4).

Having established an appropriate mathematical system that can generate the trajectory types observed from the data, step 3 of the dynamical systems approach consists of analysing the behavior of the mathematical system. In particular, *sensitivity analysis* helps to determine how changes in individual parameter values affect the trajectories and hence helps to identify the key system parameters to be used as phenotypes in subsequent genetic analyses. Bifurcation *theory* can be applied to identify the parameter regions corresponding to the different trajectory types (Steindl and Feichtinger, 2004; Blyuss and Gupta, 2009). Finally, *stability theory* helps to determine the stability of trajectories under small perturbations of the initial conditions (represented, for example, by different challenge doses, different host immunity at the start of the infection, or different performance levels prior to challenge) (Blyuss and Gupta, 2009; Taylor and Carr, 2009). Overall, these techniques will generate a thorough understanding of the trajectories that can be generated in theory and will specify the range of system parameter values corresponding to desired trajectories.

The last step in the dynamical systems approach focuses on obtaining values for the model parameters for the population of animals in consideration. This can be achieved by fitting the mathematical model established in step 2 to actual data. Recent advances in Bayesian inference methods (e.g., Savill et al., 2009; Miller et al., 2010) have demonstrated that realistic estimates for system parameters can be obtained from relatively sparse sets of data.

Once estimates for the key system parameters for every individual are obtained, one can use these as phenotypes for conventional quantitative genetic analyses to identify to what extent the trajectories are under host genetic influence. Using model parameters has several benefits over using actual measurements as phenotypes for subsequent analyses. For example, the information provided by many measurements can be summarized into few parameters, measurement error and noise become “averaged out,” and with adequate data the corresponding trajectories over time are fully specified, even if available data only relate to a fragment of the full trajectory. Combining results from quantitative genetic analyses with the understanding obtained from the analysis of the system behavior would give new insights into individual impacts of infection on performance. In turn this should help (1) to predict selection responses in terms of infection severity and impact on performance and (2) to identify new informative traits (i.e., the key parameters with a large genetic influence) to be targeted for genetic improvement.

## Discussion

Host resistance and tolerance describe the ability of a host to control infection severity and impact on performance. These traits encompass a variety of immune-pathological processes, which are highly dynamic in nature. Thus, the expression of host genetic resistance and tolerance is expected to vary considerably over time. Hitherto, quantitative genetics has treated host resistance and tolerance as static traits. As such, most empirical estimates of both characteristics to date are based on cross-sectional measurements of indicator traits obtained at a specific point in time. However, this static approach applied to dynamic traits not only raises questions about whether the obtained resistance and tolerance parameter estimates are stable over the time course of infection, but also lead to severe estimation bias if field data used as individual records refer to different infection stages across individuals (Bishop et al., 2012; Doeschl-Wilson et al., 2012). In the context of tolerance, cross-sectional measurements impose the additional limitation that a tolerance phenotype can only readily be estimated at the group rather than at the individual level.

Longitudinal measurements of performance and resistance traits (i.e., individual records taken at successive times) are routinely collected for a number of species and diseases (e.g., mastitis or gastro-intestinal parasitism in ruminants). Recording frequency of health traits may even increase if the benefits to livestock production were clearly demonstrated. We have shown in this article that longitudinal measurements provide the opportunity to quantify tolerance at the level of individuals, and may produce new informative phenotypes that describe the interactive effects of host resistance and tolerance over time. In particular, we have shown how, in principle, individual tolerance estimates could be obtained within the conventional random regression framework, and introduced a non-conventional dynamical systems approach to generate new phenotypes describing impacts of infectious pathogens on hosts. The pros and cons of these approaches will be discussed below, but it should first be noted that both approaches rely on the following conditions:

- Both host performance and pathogen burden can be measured repeatedly over the time period under consideration for each individual.
- The performance trait is such that the phenotype for individuals in the absence of pathogen challenge is either known or can be inferred for the time period over which tolerance is estimated.
- Other factors influencing performance throughout the time period of infection, in addition to pathogen challenge, can be properly accounted for.

Random regression models have proved to be a powerful tool for quantitative genetic analyses, not only for linear models but also for measures that represent curves (Meyer and Kirkpatrick, 2005). Such models are particularly attractive for quantitative genetic analyses of host resistance and tolerance as they can be readily accommodated in the conventional multivariate linear mixed model framework of quantitative genetics. Consequently, established methods can be used for estimating genetic parameters associated with host resistance and tolerance, and for genetic evaluations. However, in addition to a requirement for large datasets, a major potential drawback of using random regression models for tolerance is that these models require an explicit mathematical expression describing the relationship between host performance and pathogen burden. Meyer and Kirkpatrick (2005) demonstrated that the random regression approach can be applied to a wide range of non-linear functions, as long as the model is linear in the regression coefficients. However, as both host performance and pathogen burden vary over time and are mediated by the host immune response, it may not always be possible to express this relationship by a suitable function. In fact, even the relatively simple model described by Equations (5) and (6), points to two major issues that may need to be dealt with: firstly, the independent variable, pathogen burden itself is a function of time, and may change (increase, decrease, or both) over time. The independent variables of random regression models are however typically continuous and strictly monotonic (e.g., time, age, etc.). Secondly, the dynamic systems approach revealed that the relationship between host performance and pathogen burden over the time course of infection may not be representable as a mathematical function, but rather as a mathematical relation. Mathematical functions are a subset of relations for which every value of an independent variable (here pathogen burden) corresponds to a unique value of the dependent variable (here performance). As illustrated by the trajectories in Figure 1, this may not be the case as a particular value of pathogen burden may correspond to two or more performance trait values. The properties and applicability of the random regression approach has yet to be fully investigated.

The trajectory approach relaxes the stringent assumption of random regression models that the relationship between host performance and pathogen burden can be adequately represented by a mathematical function. Instead, a simple classification of the data trajectories into one of nine categories may provide a useful categorical phenotype for subsequent genetic studies. Furthermore, trajectories would enable the introduction of powerful tools from dynamical systems theory into quantitative genetics. According to dynamical systems theory, performance is related to pathogen burden by a system of differential equations that describes the dynamic host–pathogen interactions affecting infection severity and impact. The close affinity of the system's parameters to the underlying biological processes makes these parameters attractive phenotypes for subsequent genetic analyses. Although rarely used in quantitative genetic analyses, differential equation models appear a promising tool for handling dynamic processes within the conventional quantitative genetics framework. Integration of these more complex mathematical models into genetic analyses, as outlined by the different steps above, may enhance our understanding of the key processes that determine infection severity and reduction in performance and simultaneously provide valuable insight about the host genetic influence on these.

Dynamical systems theory is well-established in mathematics for analyzing the full spectrum of patterns produced by a complex dynamical system. They have proved useful to study infection processes within individual hosts and in populations (e.g., Blyuss and Gupta, 2009; Taylor and Carr, 2009). In the context of genetic improvement programmes, dynamical systems theory would not only provide new phenotypes, but also help to specify potential improvement targets. In particular, it can be used to determine which types of pathogen burden–performance trajectories could be produced in principle and how much shift (i.e., genetic gain) in the parameter values is required to achieve desirable trajectories.

As outlined above, the individual building blocks needed for adopting resistance-tolerance trajectories into breeding programmes already exist. There are, however, several challenges associated with the dynamical systems approach, of which perhaps the biggest one is to identify an appropriate model that reproduces the essential features observed from the data. There is a vast literature on dynamic host–pathogen interaction models [see e.g., reviews by Louzoun (2007); Mata and Cohn (2007); Doeschl-Wilson (2011)], ranging from simple models such as the one presented here [Equation (7)], where pathogens and immune response are summarized as single entities (e.g., Antia et al., 1996; Restif and Koella, 2004; Doeschl-Wilson et al., 2009), to highly complex models comprising a large number of differential equations with many parameters (e.g., Marchuck et al., 1991; Kosmrlj et al., 2010). For the purpose of quantitative genetic analyses, simple models requiring fewer parameters and thus giving rise to fewer phenotypic traits are more attractive than complex models. Nevertheless, it remains to be tested whether a relatively simple model can adequately reproduce the main features of the pathogen burden–performance trajectories emerging from the data. Furthermore, if an appropriate model can be identified, the next challenge that arises is to fit the model to existing data. Bayesian methods have proved powerful in providing reliable parameter estimates for differential equation models (e.g., Girolami, 2008; Savill et al., 2009; Miller et al., 2010), but to the best of our knowledge have not been applied to the large data sets needed for quantitative genetic analyses. Further optimization of the computational algorithms may be necessary for efficiently handling the large amount of data usually required for genetic analyses.

Finally, it should be noted that the trajectory approach can be adapted to field data, as the methodology doesn't require individuals to be at the same stage of infection nor does it require prior knowledge of the time of onset of infection. This approach may thus provide a means to capture tolerance of animals to infections under natural rather than experimental pathogen challenge, which has been difficult up to now (Doeschl-Wilson et al., 2012).

## Conclusions

Host genetic resistance and tolerance to infectious pathogens are highly desirable targets for genetic improvement. Up to now genetic analysis of host tolerance has been hindered by the lack of appropriate methods to obtain reliable tolerance phenotypes, in particularly at the level of individuals. We have outlined two alternative approaches to fill this gap, a statistical random regression approach and a mathematical dynamical systems approach. We have shown that random regression models provide a means of extending the methodology of quantifying group tolerance to the individual level. However, application of these models in practice comes with strict data requirements and depends on whether the relationship between within-host pathogen burden and performance can be adequately represented by a mathematical model that is linear in its regression coefficients. Mathematical dynamical systems theory offers a promising alternative to the statistical models currently used in quantitative genetics, as it captures the dynamic interaction of host resistance and tolerance mechanisms throughout the infection and provides static parameters amenable for genetic analyses. It builds upon performance-pathogen burden trajectories that can be derived from repeated pairwise observations of host performance and pathogen burden over the time course of the infection. Future studies are warranted to test the theoretical concepts introduced here with simulated and real data.

### Conflict of interest statement

The authors declare that the research was conducted in the absence of any commercial or financial relationships that could be construed as a potential conflict of interest.
